# The Gut Mycobiome in Inflammatory Bowel Disease: Reframing *Candida* as a Signal of Ecosystem Disruption

**DOI:** 10.3390/jof12080566

**Published:** 2026-08-01

**Authors:** Sandro Mereu, Elettra Merola, Giovanni Mario Pes, Maria Pina Dore

**Affiliations:** 1 Dipartimento di Medicina, Chirurgia e Farmacia, University of Sassari, Viale San Pietro 43, 07100 Sassari, Italy; s.mereu6@studenti.uniss.it (S.M.); emerola@uniss.it (E.M.); gmpes@uniss.it (G.M.P.); 2 Department of Medicine, Baylor College of Medicine, One Baylor Plaza Blvd, Houston, TX 77030, USA

**Keywords:** inflammatory bowel disease, *Candida albicans*, gut mycobiome, dysbiosis, cross-kingdom interactions, microbial ecosystem, fungal colonization

## Abstract

Background: Growing interest in the gut mycobiome has renewed focus on *Candida* spp. in inflammatory bowel disease (IBD). Yet, fecal detection remains difficult to interpret, given its uncertain relationship with intestinal inflammation. This narrative review synthesizes historical, mechanistic, observational, and interventional evidence to distinguish colonization, relative abundance, mucosal invasion, virulence transitions, and potential contributions to IBD. Methods: PubMed, Scopus, the Cochrane Library, and Google Scholar were searched, focusing on human studies and major mechanistic or interventional contributions. The final synthesis included 43 studies, 41 research articles, and two historical monographs. Results: Fungal alterations in IBD are heterogeneous and method-dependent across studies. Longitudinal cohorts have associated increased relative abundance of the genus *Candida* with active disease, but findings have not been consistently replicated across populations. Experimental evidence suggests that fungal expansion, morphology, and strain-specific virulence may trigger inflammation in selected settings, although stool sequencing cannot establish viability, invasion, morphology, or causality. Antifungal therapy, fecal microbiota transplantation, and nutraceutical approaches may modify microbial or inflammatory markers, but consistent clinical benefits remain unproven. Conclusions: Fecal detection of *Candida* spp. should generally be interpreted as a context-dependent signal of disrupted bacterial–fungal–immune interactions, not as evidence of infection or a compelling indication for antifungal treatment.

## 1. Introduction

### 1.1. Historical Framing

Inflammatory bowel diseases (IBDs), comprising ulcerative colitis (UC) and Crohn’s disease (CD), are chronic, relapsing inflammatory conditions that primarily affect the gastrointestinal tract. They are multifactorial disorders with a growing global burden and a substantial clinical and socioeconomic impact. Chronic diarrheal disorders with occasional blood were already described by Hippocrates in the 4th century BC, centuries before modern nosology [1,2]. However, a more specific categorization of idiopathic colitis emerged only in the nineteenth century, when Sir Samuel Wilks reported the case of Miss Bankes, a young woman suffering from chronic bloody diarrhea and severe wasting. At autopsy, Wilks found continuous ulceration and inflammation of the colon, not attributable to tuberculosis or infectious dysentery, and defined it as an autonomous pathological entity, later termed ulcerative colitis [3]. Several decades later, in 1932, Burrill B. Crohn, Leon Ginzburg, and Gordon Oppenheimer described patients with chronic inflammation and ulceration of the terminal ileum that could not be explained by known infectious conditions, a disorder they termed regional ileitis, subsequently known as CD [4]. During the late nineteenth and early twentieth centuries, at the dawn of the microbiological era, scientific efforts were primarily directed toward identifying an infectious cause for these disorders [5]. The microscopic lesions observed in UC, first detailed by Hale-White in the 1880s and later by Bargen in the 1920s, were interpreted within this paradigm. Their studies described continuous mucosal ulceration, dense inflammatory infiltrates, and loss of epithelial integrity in UC. In contrast, Crohn and colleagues [4] identified the characteristic transmural inflammation and non-caseating granulomas of regional ileitis.

### 1.2. Conceptual Framing

Despite decades of attempts, no single pathogen could consistently account for these chronic conditions. Repeated failures to identify an infectious etiology gradually undermined the prevailing paradigm and led to the reinterpretation of UC and CD as chronic inflammatory disorders with multifactorial pathogenesis rather than purely infectious processes [6]. Attention consequently shifted from the search for a contagious agent to host susceptibility, immune dysfunction, and intestinal microbiota. Large population datasets indicate that patients with IBD, particularly older adults and those receiving immunosuppressive therapy, experience an increased incidence of infection. In a cohort of more than 63,759 patients with IBD, infection risk was higher among older individuals and those exposed to corticosteroids, anti-TNF-α agents, or immunomodulators [7]. Candidiasis was one component of this burden, suggesting that clinically recognized fungal disease frequently arises in the context of host susceptibility and treatment-related immunosuppression. However, this does not preclude direct fungal pathogenicity once mucosal or invasive disease is established. IBD itself arises from the interplay of host genetics, immune dysregulation, environmental exposures, and the gut microbiota [8,9]. Genetic variants affecting innate immune and autophagy pathways, including NOD2 and ATG16L1, may impair microbial sensing and epithelial defense. Diet, antibiotics, smoking, and early-life exposures further modify disease risk, partly by reshaping the gut microbiota [10]. These observations provide the basis for examining bacterial, fungal, viral, metabolic, and host interactions in intestinal inflammation [9,10,11].

Despite growing interest in the gut mycobiome, current evidence is fragmented and often interpreted within an infection-centered framework. We therefore aimed to provide an integrated ecological perspective by synthesizing historical, mechanistic, and clinical evidence to clarify the biological and clinical significance of *Candida* spp. in IBD. Moreover, whether current evidence supports routine stool testing or antifungal treatment was addressed based on the levels of evidence underlying proposed cross-kingdom mechanisms. Finally, a clinically testable working hypothesis for the role of *Candida* spp. in IBD was formulated.

## 2. Materials and Methods

This article is a narrative review integrating historical, clinical, microbiome, and immunological data on the relationship among *Candida* spp., the gut mycobiome, and IBD. A non-systematic literature search was performed in PubMed, Scopus, the Cochrane Library, and Google Scholar. The core strategy combined (“Inflammatory Bowel Diseases” OR “Crohn Disease” OR “Ulcerative Colitis” OR IBD) AND (“*Candida*” OR “*Candida albicans*” OR candidiasis OR fungi OR fungus OR fungal OR mycobiome). Searches were restricted to English-language, peer-reviewed publications available up to February 2026, with priority given to human observational and interventional studies. Historical, mechanistic, and preclinical studies were retained when they clarified IBD pathogenesis, bacterial-fungal interactions, host immune responses, or fungal virulence. Additional sources were identified by backward citation tracking. The final narrative synthesis comprised 43 cited sources: 41 journal publications and two historical monographs. The available literature was stratified into human observational, human interventional, or mechanistic/preclinical, and conclusions were calibrated to the corresponding evidence level.

Taxonomic nomenclature was checked against current fungal and bacterial nomenclature resources, including Index Fungorum and List of Prokaryotic Names with Standing in Nomenclature.

## 3. Results

### 3.1. The Gut Microbiota: From Symbiosis to Dysbiosis

The human microbiota comprises bacteria, archaea, fungi, and viruses that colonize distinct body niches and form functionally integrated ecosystems [12]. Community composition varies substantially among individuals, whereas core metabolic functions are relatively conserved and influenced by diet, host genetics, environmental exposures, and local conditions such as luminal pH [13]. In eubiosis, microbial fermentation products, including short-chain fatty acids (SCFAs), support epithelial integrity, metabolism, and immune homeostasis [12]. Dysbiosis refers to the disruption of microbial composition and function and is consistently associated with IBD. Common bacterial findings include reduced diversity, depletion of selected Firmicutes phyla, and expansion of Proteobacteria phyla, particularly in CD [14]. Nevertheless, no single stable microbial signature has emerged, and microbiome-targeted interventions have not consistently produced durable disease modification [15]. These observations support a multi-kingdom model in which ecosystem-level interactions, rather than the presence or absence of a single taxon, influence intestinal stability [16,17].

### 3.2. The Adult Human Gut Microbiota

The bacterial component of the adult gut microbiota is dominated by the Firmicutes and Bacteroidetes phyla, followed by the Actinobacteria, Proteobacteria, and Verrucomicrobia phyla. Many members of the Lachnospiraceae and Ruminococcaceae families, along with the genera *Eubacterium* and *Faecalibacterium*, produce butyrate and contribute to epithelial energy metabolism. Bacteroidetes phylum, including *Bacteroides fragilis* and *Prevotella* spp., participates in the degradation of complex polysaccharides and metabolic cross-feeding [12,13]. Fungi constitute only a minor fraction of the gut microbiota, with approximately 10^5^–10^6^ cells/g of feces compared with 10^11^ bacterial cells/g, and eukaryotic genes representing only about 0.1% of the current gut metagenomic reference catalog [18]. The phylum Ascomycota commonly predominates and includes *Saccharomyces cerevisiae*, *Candida albicans*, and *Aspergillus* spp., whereas *Malassezia* spp. belong to the phylum Basidiomycota. *Saccharomyces*, *Candida*, and *Malassezia* are among the genera most frequently detected in healthy adult stool, but no universal core gut mycobiome has been established because fungal profiles vary with diet, geography, sampling site, and analytical method [18,19]. In IBD, some cohorts have reported a higher Basidiomycota-to-Ascomycota division ratio and increased relative abundance of *C. albicans*, whereas others have not reproduced these taxonomic patterns [18,19]. The intestinal virome contains abundant bacteriophages, including members historically classified within Caudovirales and the Microviridae family [20,21]. The archaeome, including *Methanobrevibacter smithii* and *Methanosphaera stadtmanae*, participates in hydrogen transfer and fermentation, and altered archaeal profiles have also been reported in IBD [18].

### 3.3. Candida albicans: From Commensalism to Opportunism

*Candida albicans* is a common human commensal and an opportunistic pathogen whose behavior depends on host, microbial, and local tissue conditions [22,23]. Several distinct states must be separated. Fecal detection indicates luminal presence but does not demonstrate mucosal invasion. Relative abundance is a compositional sequencing measure and does not establish absolute fungal load. Mucosal invasion requires tissue-level evidence, whereas virulence-state transitions involve morphology, gene expression, and strain-specific traits. These states should not be treated as interchangeable. *Candida albicans* can alternate among blastospores, pseudohyphae, true hyphae, and stress-associated forms, with different biological implications [23,24] (Table 1).

- Blastospores: round or oval budding cells that commonly predominate during commensal luminal colonization;
- Pseudohyphae: elongated chains of budding cells with constrictions at the septa, representing a transitional filamentous morphology that may contribute to adhesion and tissue interaction;
- True hyphae: long filamentous cells without septal constrictions that can express adhesins, hydrolytic enzymes, and candidalysin and can penetrate tissue in experimental and mucosal infection settings;
- Chlamydospores: thick-walled stress-associated cells whose relevance to human intestinal colonization and IBD remains uncertain.

**Figure 1:**
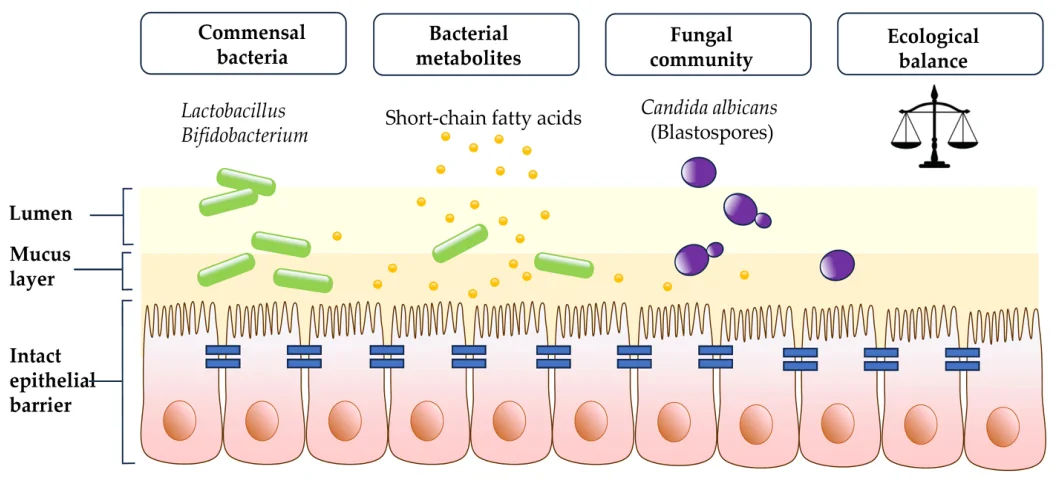
Proposed bacterial-fungal-immune interactions in intestinal homeostasis. Commensal bacteria and their metabolites may help maintain *Candida albicans* in a predominantly blastospore state, preserve epithelial barrier integrity, and support antifungal immune surveillance through pathways including Dectin-1/CARD9.

Morphological transitions are reversible and are regulated by temperature, pH, CO_2_, nutrients, and intracellular signaling pathways, including cAMP-PKA and MAPK [24,25]. Hyphal forms can express hydrolases and candidalysin, which damage epithelial barriers and activate inflammatory responses in experimental and mucosal infection models [26,27,28]. Disruption of bacterial or immune constraints may favor filamentation and virulence-associated programs [27,28]. However, sequencing-based detection of *C. albicans* in stool cannot determine whether organisms are viable, whether blastospores or hyphal forms predominate, or whether tissue invasion is present. These distinctions are essential when extrapolating mechanistic data to human IBD (Figure 1).

**Figure 2:**
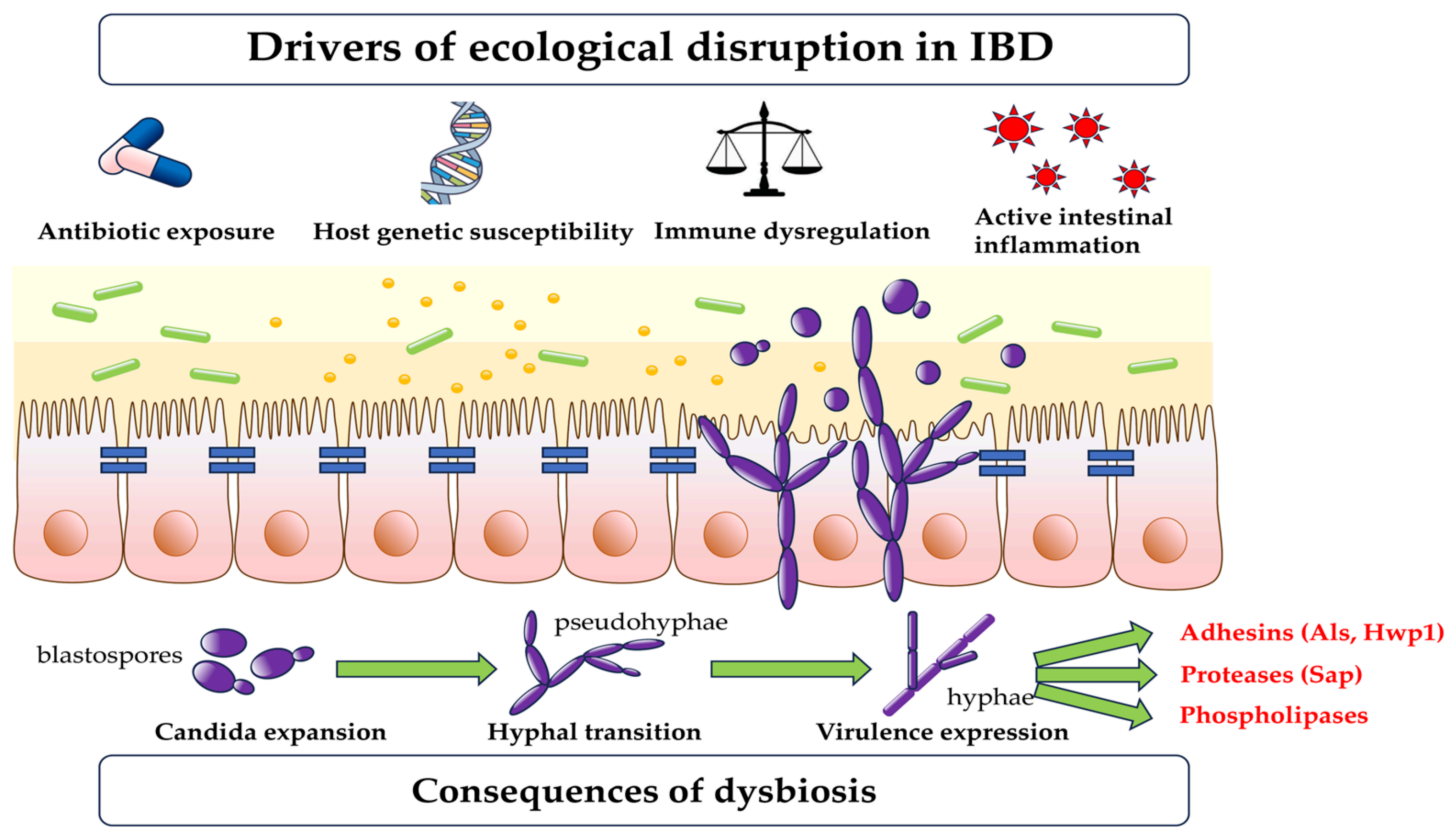
Proposed ecosystem disruption and fungal dysbiosis in IBD. Antibiotic exposure, host susceptibility, immune dysregulation, and active inflammation may alter bacterial–fungal–immune interactions, favor an increased relative abundance of *Candida albicans*, and, under selected conditions, virulence-associated morphology. These changes may contribute to barrier dysfunction and immune activation, but the direction and causal importance of each pathway in human IBD remain unresolved.

Mucosal immune surveillance is a critical determinant of fungal containment. Recognition of fungal cell-wall components through Dectin-1 and CARD9 can activate IL-17- and IL-22-related responses that support barrier defense [29,30]. Experimental deficiency of CARD9 or Dectin-1 worsens colitis, whereas pharmacologic blockade of IL-17 can aggravate intestinal inflammation and increase susceptibility to fungal infection, illustrating that Th17-mediated immunity can be both protective and inflammatory depending on context [29]. Bacterial–fungal effects on morphology have largely been demonstrated in experimental systems: selected *Lactobacillus* and *Bifidobacterium* genera or their metabolites can inhibit *C. albicans* filamentation and biofilm formation [28,31], whereas antibiotic-associated depletion of bacterial competitors and SCFAs can facilitate increased fungal load and filamentation in models [32]. Experimental fungal colonization can also shape mucosal immune education [30]. The extent to which each of these mechanisms operates in individual patients with IBD remains uncertain (Figure 2).

### 3.4. Cross-Kingdom Signaling and Microbial Cooperation

Cross-kingdom signaling provides plausible mechanisms by which fungi could contribute to intestinal inflammation, but the underlying evidence spans in vitro systems, animal models, nonintestinal infection models, and human correlation studies. In experimental systems, peptidoglycan fragments and N-acetylglucosamine (GlcNAc can) activate the *C. albicans* cAMP-PKA pathway and induce hyphal growth, whereas lactic acid produced by selected *Lactobacillus* spp. can suppress Ras1-Efg1 signaling and favor the yeast state [28,31]. Farnesol-mediated interference with bacterial quorum sensing, ethanol-associated enhancement of *Acinetobacter baumannii* virulence, and mixed-species biofilms involving *C. albicans*, *Escherichia coli*, and *Enterococcus faecalis* have also been described, primarily in non-IBD or experimental settings [17,32]. Similarly, reciprocal immune-metabolic feedback involving fungal cell-wall components, bacterial metabolites, Dectin-1/CARD9 signaling, and Th17 differentiation remains mechanistically compelling but incompletely validated in human IBD [28,29,31]. Human multi-omics studies have identified inter-kingdom correlations, including relationships between the genus *Candida* and *Bacteroides thetaiotaomicron* and between *Saccharomyces* genus and branched-chain fatty acids [33,34,35]. Such correlations support network-level hypotheses but do not establish direct microbial interaction or causality. Accordingly, this section should be interpreted as a map of candidate mechanisms rather than evidence that these processes uniformly occur in the inflamed human intestine.

### 3.5. Human Observational Evidence: Disease Association, Activity, and Host Context

Human studies support an association between IBD and altered fungal community structure, but taxonomic findings are heterogeneous and vary by cohort, geography, sampling site, treatment exposure, and analytical method [19,29,36]. Sokol et al. reported increased relative abundance of *C. albicans*, a higher *Basidiomycota*-to-*Ascomycota* ratio, and reduced *S. cerevisiae* in IBD compared with healthy controls using ITS2 sequencing [19]. By contrast, Frau et al. [34] integrated bacterial, fungal, and metabolic data from two independent cohorts and identified cohort- and niche-specific fungal signatures rather than universal ones. The genus *Candida* showed a negative correlation with *B. thetaiotaomicron* in one cohort, whereas the genus *Malassezia* and the order Eurotiales were enriched in selected CD samples. These findings are consistent with a context-dependent network signal but cannot determine whether fungal changes are a cause, a consequence, or an amplifier of inflammation.

Serologic responses provide related but distinct information. Anti-*Saccharomyces cerevisiae* antibodies (ASCAs) are associated with CD but represent a heterogeneous response to overlapping yeast glycans and host factors rather than a pathogen-specific signature [37]. Mechanistic work on Dectin-1/CARD9 and Th17 pathways further supports the biological relevance of antifungal immunity [38], but does not establish that fecal *Candida* spp. abundance is an independent disease driver. Bahr et al. evaluated invasive fungal infections among 33,536 patients initiating IL-17 or IL-23 inhibitors and observed 125 events (3.7 per 1000 patient-years), most commonly esophageal candidiasis; the remaining infections included *Histoplasma* spp., *Aspergillus* spp., and other filamentous species [39]. This was not a gut mycobiome study and should not be used to infer luminal fungal ecology. It does, however, reinforce the clinical distinction between fecal colonization and mucosal or invasive fungal disease.

Disease–activity associations have been reported in individual longitudinal cohorts. In a large UC cohort, Jangi et al. found that relative abundance of the genus *Candida* was approximately 3.5-fold higher during clinical activity than during remission. In the longitudinal subset, patients whose clinical status changed showed parallel shifts in the relative abundance of the genus *Candida*, whereas those with stable status had more stable profiles [40]. These data support a temporal association in that population but do not establish directionality or causation. Similar associations have not yet been consistently replicated across populations, sampling methods, and analytical platforms [36]. Table 2 summarizes the principal human studies discussed in this review and separates observational association from interventional evidence.

### 3.6. Interventional and Microbiota-Restorative Evidence

Interventional studies provide stronger evidence of biological responsiveness but have not established routine clinical benefit. Jena et al. randomized 61 patients with active UC and detectable stool *Candida* spp. to fluconazole or placebo. The prespecified primary clinical endpoint was not met (16.1% vs. 3.3%; *p* = 0.19), although histologic healing and fecal calprotectin reduction favored fluconazole in secondary analyses [35]. These findings suggest that antifungal treatment can influence inflammatory or mucosal markers in a selected population, but they do not prove that the effect results from restoration of bacterial–fungal balance or that antifungal therapy improves patient-important outcomes.

In a prospective study of encapsulated fecal microbiota transplantation (FMT), 12 of 21 evaluable patients with active UC achieved clinical remission. Responders showed a reduced relative abundance of *Candida* spp. and *Debaryomyces hansenii*, along with donor-like changes in *Saccharomyces*, *Kazachstania*, and *Lachancea* spp. [33]. Because the study was small and uncontrolled, fungal restructuring may have been a marker of successful engraftment or clinical improvement rather than its cause. The FMT changes multiple microbial and metabolic compartments simultaneously, preventing attribution of benefit to fungi alone.

Nutraceutical approaches remain preclinical. Fucoidan has been reported to modify bacterial taxa, suppress *C. albicans* in experimental colitis models, and affect NF-κB/MAPK signaling and epithelial barrier markers [41,42]. These observations illustrate a possible ecosystem-restorative strategy but cannot currently support clinical recommendations. Overall, antifungals, FMT, and nutraceutical interventions should be regarded as investigational probes of fungal–microbial biology rather than validated mycobiome-directed therapies.

## 4. Discussion

### 4.1. Clinical Implications: Colonization Is Not Candidiasis

Current clinical guidance addresses the genus *Candida* as a cause of oropharyngeal, esophageal, or invasive candidiasis in susceptible patients and does not recommend routine stool testing for *Candida* spp. in UC or CD [43]. This distinction is consistent with the available evidence. Fecal detection or increased relative abundance does not demonstrate viable overgrowth, mucosal invasion, or systemic infection and has no validated quantitative threshold or reproducible predictive value for IBD outcomes [36]. Accordingly, an isolated positive stool result should not be treated as an independent indication for antifungal therapy.

Classical mucosal or invasive fungal disease remains a distinct clinical entity and requires investigation and treatment in accordance with established infectious disease criteria. Conversely, in the absence of tissue involvement or systemic candidiasis, antifungal treatment for luminal colonization alone is not supported. The fluconazole trial in active UC demonstrated changes in selected secondary biological outcomes but did not meet its primary clinical endpoint [35]. Mycobiome assessment should therefore remain a research tool, including in longitudinal cohorts, FMT studies, and multi-omics investigations, rather than a component of routine IBD monitoring.

### 4.2. Methodological Limitations of Gut Mycobiome Research

Interpretation of the gut mycobiome is constrained by methodological factors that are more consequential for fungi than for abundant bacterial communities. Fungal biomass and sequence counts are low, making results vulnerable to reagent and environmental contamination, stochastic amplification, and loss of signal. Mechanical cell disruption; DNA extraction protocol; choice of ITS1, ITS2, 18S, or metagenomic sequencing; primer bias; sequencing depth; taxonomic database quality; and bioinformatic thresholds can each alter the taxa detected. The recent systematic review of case–control studies found substantial heterogeneity in these domains and inconsistent species- and genus-level results across studies [36].

Biological sampling creates additional uncertainty. Stool and mucosal biopsy samples represent different niches, and fecal fungi may include transient dietary or environmental organisms rather than stable colonizers. Relative-abundance data are compositional: an apparent increase in *Candida* spp. can result from changes in other taxa without an increase in absolute fungal load. Standard DNA sequencing generally cannot determine viability, distinguish blastospore from hyphal morphology, identify spatial proximity to the epithelium, or demonstrate tissue invasion. Medication exposure, diet, geography, disease location, inflammation, antibiotics, and immunosuppression are major confounders [29]. These limitations explain why colonization, abundance, morphology, invasion, and causal contribution must be reported as separate concepts.

### 4.3. Research Priorities and Working Hypothesis

The most extensively investigated area has been cross-sectional compositional profiling of fecal fungal communities in IBD compared with healthy controls. By contrast, major gaps remain in longitudinal sampling, stool–mucosa concordance, absolute fungal quantification, viability assessment, morphology and spatial localization, strain-level virulence, integration with bacterial and metabolic function, and controlled intervention studies. Future research should combine standardized collection and extraction, contamination controls, quantitative assays, culture or viability-informed methods, mucosal imaging or histology where appropriate, and prospectively defined clinical outcomes. Adequate stratification by treatment, antibiotic exposure, diet, disease phenotype, and activity is essential [36].

Based on current evidence, we propose the following working hypothesis: isolated fecal detection or increased relative abundance of *Candida* spp. in IBD most often functions as a context-dependent signal of disruption within the bacterial–fungal–immune ecosystem rather than as evidence of an autonomous infection. This challenges an infection-centered interpretation of stool findings but does not imply that fungi are biologically inert. In selected patients, increased absolute biomass, strain-specific virulence, hyphal transition, biofilm formation, or impaired antifungal immunity may amplify an already established inflammatory environment. Consequence and contributor are therefore not mutually exclusive.

Microbiota-directed strategies remain investigational. FMT, antifungal therapy, and nutraceutical interventions can alter fungal or inflammatory measures [33,34,35], but no intervention has yet demonstrated reproducible, fungus-specific, disease-modifying benefit in routine IBD care. The immediate objective should be to determine which fungal signals are transient markers, which identify a biologically relevant host-microbial state, and whether any such state predicts response to established or adjunctive therapy.

## 5. Conclusions

The available evidence supports a working hypothesis rather than a settled causal model. In most patients with IBD, isolated fecal detection or increased relative abundance of *Candida* spp. should be interpreted as a context-dependent signal of disrupted bacterial-fungal-immune interactions. It should not, by itself, be considered as evidence of candidiasis or an indication for anti-fungal treatment. This ecosystem-based interpretation does not exclude the possibility that increased absolute fungal biomass, virulence-state transitions, mucosal proximity, or strain-specific traits amplify inflammation in selected patients. Human studies reporting associations with disease activity remain heterogeneous, and interventional studies have not demonstrated consistent clinical benefit. Routine stool testing and mycobiome-directed treatment, therefore, remain unsupported outside research settings and in established cases of mucosal or systemic fungal disease. Future studies should use standardized longitudinal sampling, absolute and viability-informed quantification, tissue-level assessment where appropriate, and integrated multi-omics designs to test whether specific fungal states have diagnostic, prognostic, or therapeutic value.

## Figures and Tables

**Table 1:** Morphological forms of *Candida albicans* and major environmental and immunological regulators of the blastospore–hyphae transition.

Morphology	Key Feature	Environmental Regulators	Interpretation in IBD
Blastospores	Budding commensal morphology; usually non-invasive during luminal colonization	SCFAs ^1^, lactic acid, acidic or fermentative conditions	Luminal colonization; clinical significance depends on host and tissue context
Pseudohyphae	Transitional filamentous morphology	Nutrient limitation and selected stress signals	May contribute to adhesion and mucosal interaction; IBD-specific role remains uncertain
True hyphae	Filamentous morphology with tissue-invasive potential in experimental and mucosal infection settings	Neutral-to-alkaline pH, elevated CO_2_, temperature, PGN ^2^/GlcNAc ^3^, and reduced SCFAs	Potential barrier injury and inflammatory signaling; not inferable from stool sequencing
Chlamydospores	Stress-associated survival form	Adverse environmental conditions	Role in the human intestinal mycobiome and IBD remains uncertain

^1^ Short-chain fatty acids; ^2^ peptidoglycan; ^3^ N-acetylglucosamine (GlcNAc).

**Table 2:** Summary of major human studies informing the interpretation of the gut mycobiome and genus *Candida* in IBD.

Study	Design/Population	Assessment	Main Finding	Key Limitation/Evidence Level
Sokol et al. (2017) [19]	Case–control; IBD and healthy controls	Fecal ITS2 sequencing	Higher *Candida albicans* relative abundance, higher Basidiomycota/Ascomycota division ratio, and lower *Saccharomyces cerevisiae* in IBD	Cross-sectional and compositional; association does not establish causality
Frau et al. (2021) [34]	Two multi-omics cohorts including CD, UC, and controls	Stool/biopsy fungal, bacterial, and metabolic profiling	Cohort- and niche-specific fungal signatures; inter-kingdom correlations involving *Candida* and *Bacteroides* genera	Heterogeneous cohorts and low fungal yield in some biopsy samples
Jena et al. (2022) [35]	Randomized placebo-controlled trial; active UC with *Candida*-positive stool	Stool detection plus clinical, histologic, and biomarker outcomes	Primary clinical endpoint was not met; histologic healing and calprotectin improved in secondary analyses	Small randomized subgroup, concomitant therapy, short follow-up; clinical efficacy unconfirmed
Chen et al. (2022) [33]	Prospective encapsulated fecal microbiota transplantation study; active UC	Longitudinal fungal profiling	Responders showed donor-like fungal restructuring and reduced *Candida* spp. and *Debaryomyces hansenii*	Small uncontrolled cohort; association with response does not prove mediation
Jangi et al. (2024) [40]	Large cross-sectional UC cohort with longitudinal subset	Fecal ITS2 sequencing	The genus *Candida* relative abundance was higher during clinical activity and changed with status in a subset	Clinical activity based mainly on symptoms; genus-level relative abundance and limited longitudinal sample
Bahr et al. (2024) [39]	Large real-world cohort initiating IL-17/IL-23 inhibitors	Administrative data on invasive fungal infection	Low absolute incidence of invasive fungal infection; esophageal candidiasis predominated	Not a gut mycobiome study; informs infection risk rather than luminal ecology

## Data Availability

data were created or analyzed in this study. Data sharing is not applicable to this article.

## Abbreviations

The following abbreviations are used in this manuscript:

ASCA	Anti-*Saccharomyces cerevisiae* antibody
cAMP-PKA	Cyclic adenosine monophosphate-protein kinase A
CD	Crohn’s disease
ECCO	European Crohn’s and Colitis Organisation
FMT	Fecal microbiota transplantation
IBD	Inflammatory bowel disease
IFI	Invasive fungal infection
MAPK	Mitogen-activated protein kinase
GlcNAc	N-acetylglucosamine
PGN	Peptidoglycan
SCFAs	Short-chain fatty acids
UC	Ulcerative colitis
